# Who funds the WHO Foundation? A transparency analysis of donation disclosures over the first 3 years of its operation

**DOI:** 10.1136/bmjgh-2025-018932

**Published:** 2025-07-23

**Authors:** Nason Maani, Emily R Adrion, Jeff Collin

**Affiliations:** 1 Global Health Policy Unit, School of Social and Political Science, The University of Edinburgh, Edinburgh, UK; 2 Public Health, Environments and Society, London School of Hygiene and Tropical Medicine, London, UK

**Keywords:** Global Health, Public Health

## Abstract

**Introduction:**

The WHO Foundation (WHOF) was established in 2020 with the stated aim of broadening the donor pool to the WHO. Academics and civil society actors raised concerns regarding the potential nature and source of contributions from commercial actors with a conflict of interest, particularly regarding the central role of commercial determinants of health in contemporary global health challenges. However, to date, little is known regarding the nature of donations to the WHOF. This analysis sought to examine the transparency and patterns of donations received by the WHOF based on the Foundation’s own disclosures.

**Methods:**

Data was extracted from funding disclosures data made available by the WHOF on its transparency page. Donations were analysed descriptively by year, donor type, amount and earmarked programme. Sankey diagrams were constructed to illustrate the magnitude and flow of funds by donor type to the specific WHO programmes for which the donated funds were earmarked. Levels of transparency were assessed for each reporting period based on the A–E ‘Who Funds You’ transparency scale used by Open Democracy.

**Results:**

Since its launch until the end of 2023, the Foundation disclosed total donation receipts of US$82 783 930 overall, of which US$39 757 326 (48%) was characterised as anonymous donations over US$100 000. The proportion of anonymous donations over US$100 000 increased year on year, alongside named donations from charitable foundations such as the Bill and Melinda Gates Foundation, social media companies, medical device companies, banking/finance and pharmaceutical companies. The largest proportion was earmarked for ‘WHO Foundation Operational Support’. The WHOF was assessed a ‘B’ transparency rating for the first reporting period, falling to a ‘D’ (less than 50% of donations by value disclosed) in the 2022 and 2023 calendar years.

**Conclusions:**

This analysis finds that current levels of donor transparency are low, potentially exposing the WHOF—and by extension the WHO—to risks of perceived reputational damage or undue influence. These risks are assumed for financial contributions that, to date, have been relatively modest and follow donor, rather than WHO, priorities.

**WHAT IS ALREADY KNOWN ON THIS TOPIC**

- The WHO Foundation was established in 2020 to support WHO Programmes via donations from a wider range of sources than the WHO is permitted to accept directly. However, little is known to date regarding the transparency, scale and earmarking of such donations.

**WHAT THIS STUDY ADDS**

- This study analysed data from funding disclosures made by the WHO Foundation between May 2020 and December 2023, applying a transparency index to gauge the overall transparency of disclosures, and using Sankey diagrams to visualise earmarked donations by time period.

**HOW THIS STUDY MIGHT AFFECT RESEARCH, PRACTICE OR POLICY**

- Results show low and declining levels of transparency over time, potentially raising concerns about the level of outside influence and role of commercial interests in setting WHO priorities.

## Introduction

 The creation of the WHO Foundation (WHOF) in 2020, at the height of the COVID-19 pandemic, marked a significant escalation in a long-articulated desire by successive WHO directors-general (DGs) to widen the donor base for the WHO.^1^ The WHO itself is predominantly funded through assessed and voluntary contributions from member states and a small number of donors, most prominently the Bill and Melinda Gates Foundation. The WHOF, in contrast, explicitly seeks to solicit funding from sources that the WHO is restricted from accessing directly, including high net worth individuals and companies.^2^ In announcing the WHOF, WHO DG Dr Tedros Adhanom Ghebreyesus stated that ‘the WHO Foundation will have to fund all elements of WHO’s work and be fully aligned with our priorities’.^3^ This was echoed by Dr Thomas Zeltner, the founder and chair of the WHOF, who at launch claimed that its primary mission was ‘to address the most pressing global health challenges of today and tomorrow by raising significant new funding for WHO from non-traditional sources’.^3^ He further stated that funding from the WHOF would be given ‘as flexibly and predictably as possible’.^3^ In responses to questions at the launch event, the WHO DG argued that the WHOF would serve to increase the volume of WHO funding and contribute to additional un-earmarked resources.^3^ These claims relate to long-standing problems in securing flexible funding for the WHO, yet fail to acknowledge long-articulated critiques of approaches to global health as increasingly reflecting donor priorities rather than those of member states.^4^

Following the launch of the WHOF, an early large donation from Nestle led to concerns being raised by civil society actors^5^ regarding undue influence and potential ‘health-washing’; mirroring critiques articulated during the protracted discussions surrounding the development of WHO’s broader Framework for Engagement with Non-State Actors, notably that facilitating access by commercial actors would be inconsistent with goals of safeguarding against undue influence.^6^ This was compounded by a lack of transparency regarding donation amounts, and a series of unannounced amendments and changes to interim gift/donations policy documents on the WHOF website.^2^ At the time of writing, the current version of the WHOF gift policy sets out specific donor exclusions, yet only for tobacco and firearms manufacturers, while fossil fuel companies, alcohol producers, sugar sweetened beverage manufacturers and vaping companies, for example, are not mentioned in any form.^7^ Substantive evidence suggests that the latter companies use donations to similar initiatives as opportunities to distract or reframe product health harms,^8 9^ to complement marketing plans and to assist in wider lobbying efforts against public health regulations,^10^,^12^ including in submissions to WHO consultations.^13 14^ Donations linked to such companies have, at times, led to public outcry and the cancellation of other proposed partnerships in global health, as was the case following the announcement of a Global Fund partnership with Heineken in 2018.^15^ Such concerns remain particularly relevant in the context of the WHO considering its global remit and unique norm-setting functions, and in light of the growing interest in understanding and addressing the commercial determinants of health.

The context for addressing such concerns is further complicated by the announcement of the withdrawal of the USA from the WHO,^16^ which can be expected to heighten pressure to widen the pool of alternative donors. In parallel, announced cancellations and pauses in wider USAID funding may place additional wide-ranging demands on WHO capacity. The Center for Global Development estimates around a quarter of 2024/2025 United States Agency for International Development funding obligations have been explicitly cancelled, with only half of all contracts explicitly preserved. These include the majority of USAID funding for infrastructure, good governance, private sector competition, basic education, child and maternal health, disaster readiness, the rule of law and human rights, trade and investment and the funding of civil society.^17^

The WHOF remains central to the future financial strategy of the WHO, as evident in the WHO’s first ever ‘investment round’ in October 2024.^18^ This included the announcement of a US$50 million commitment from the WHOF via contributions from Sanofi, Boehringer Ingelheim, Novo Nordisk, TikTok, Maybelline and a range of other partners for initiatives around mental health, sleeping sickness and diabetes.^19^ Crucially, the WHOF press release announcing the pledge emphasises the Foundation’s close links to the WHO: ‘…Through its unparalleled access to WHO, the Foundation advances health equity by connecting and collaborating with visionary corporate partners to co-create solutions that have the highest impact…’ while also emphasising its own priorities linked to donor preferences including ‘…climate change, mental health, and combating medical misinformation—areas where our corporate partners are particularly passionate’.^19^ This framing is a reminder of the range of tensions and potential contradictions in such arrangements, which are framed variously as independent, as facilitating unparalleled access to the WHO, and as linking to donor (vs WHO) priorities.

The concerns around transparency raised at the launch of the WHOF included the risks of undermining established norms regarding funding transparency, or in creating confusion regarding how these norms might apply to the WHO itself, particularly as the WHOF explicitly exists to support the WHO and uses its logo.^1 20^ The WHOF states on its ‘Commitment to Transparency’ web page that it is ‘…committed to honesty, integrity, accountability and transparency in all our operations’.^21^ However, the current gift policy offers a more caveated approach, in that it will ‘…publicly acknowledge, according to its internal stewardship guidelines, contributions and counterparties unless anonymity has been requested and approved by the WHOF management’.^7^ It would appear that such anonymity is often requested, as an Associated Press article in 2023 highlighted a relative lack of transparency in overall donations to the WHOF in its first 2 years of operation, reporting that 40% of funds received were from anonymous sources. The WHOF Chief Executive Officer was quoted in the article as saying: ‘They want to be anonymous because they’re otherwise solicited or even targeted because they’re seen to be a source of wealth. And I respect that’.^22^ This interpretation of transparency is inconsistent with the norms and practices of the WHO itself, which discloses all donations to the WHO by each contributor, and their earmarked purpose, through its programme budget web portal.^23^

Underlying these tensions are concerns regarding inadequate donor transparency, in the amount of donations received and in the level of earmarking to specific projects due to the potential for conflicts of interest. Such critiques centre around networks of influence and relationships with conflicted actors,^24 25^ propagating narrow or distorted problem definitions^26^ or focusing on ineffective approaches.^27^ While assessing transparency (regarding internal processes and donations) has been identified as a key area of concern for international organisations,^28^ such analysis is complicated by a lack of an agreed common standard against which transparency can be assessed in international organisations.^29^ In think tanks specifically, which have faced similar scrutiny in relation to donor transparency and influence, there have been efforts to facilitate transparency comparisons between entities and assess changes over time,^30^ and it is possible such tools could be adapted to serve as a comparator for international organisations.

Considering the WHO’s pivotal and unique role in a range of global health processes and in engaging with a range of stakeholders, a critical assessment of the potential risks and benefits of novel funding mechanisms such as the WHOF is particularly important. Moreover, such an assessment is relevant in the context of ongoing concerns regarding transparency, efforts to address the commercial determinants of health and long-standing critiques of philanthropy-led global health initiatives. A formal analysis of donations disclosed by the WHOF will enable assessment of the extent to which its original mission—supporting all aspects of the WHO, as flexibly as possible—has been met. It will further allow reflections on transparency standards, including whether the critiques made at launch have reflected ongoing practice. To address this gap in understanding, this study seeks to examine the transparency and nature of donor contributions to the WHOF, by examining WHOF funding disclosures over the first 3 years of its operation.

## Methods

### Data collection and analysis

Data on funding disclosures from the WHOF website^21^ related to donations by year, donor type, amount and earmarked programme were extracted in June 2024. The WHOF documentation discloses donors by name, or if the donor prefers to be anonymous, categorises donations as ‘anonymous over $100 000’ or ‘anonymous under $100 000’. The original data files published by the WHOF are included in the online supplemental appendices. For the purposes of our analysis, donors were grouped by the industrial sector they predominantly operate in or, if listed as family charitable foundations, were described as such.

Excel and Stata SE V.17 were used for data compilation, data management and analysis. Descriptive statistics were generated to summarise total donations by year, donor type, amount and earmarked programme. There was no missing data, and no additional coding or data manipulation was required. Bar charts were created to summarise funding patterns. Sankey diagrams were constructed to illustrate the magnitude and flow of funds by donor type to the specific WHO programmes for which the donated funds were earmarked. The diagrams are stratified by time period according to the periods disclosed by WHOF: May 2020–December 2021, January–December 2022 and January–December 2023. Stratification is employed both to illustrate changes over time and to account for shifts in the nature of the WHOF programme portfolio between these periods. Sankey diagrams were constructed using Flourish Studio (https://flourish.studio/, 2024).

### Assessment of transparency

The level of transparency regarding donations was assessed based on the ‘Who Funds You?’ rating system used by Open Democracy to assess the funding transparency of think tanks. The scale is applied to voluntary disclosures of donations received and offers clear criteria for each transparency band (see below) based on how donation amounts are reported as well as transparency in naming donors by overall value.^31^ These features mean it offers a way of comparing disclosures by WHOF and similar organisations over time, in a way that is reproducible and allows for the consideration of all levels of donor transparency.

Under Open Democracy’s methodology, organisations are assigned a rating on an A–E scale based on transparency criteria. However, this rating system was developed primarily to examine national-level organisations with lower budgets, and a threshold for transparency on individual donations is set at £5000 under their framework. For the purposes of this study, this threshold was increased to US$100 000, to reflect WHOF as a global entity, and to remain consistent with how the WHOF itself categorises anonymous donations (classing them as either under, or over, US$100 000). The revised rating system applied in this study is detailed below:

- A (highest level):
  - Names all funders who gave US$100 000 or more in the last reported year.
  - Declares exact amount given by each funder.
- B
  - Names at least 85% of funders (by value) who gave US$100 000 or more.
  - Groups funders into precise funding bands (organisations that use broad funding bands may be eligible for a B rating if they name all funders).
- C
  - Names at least 50% of funders (by value) who gave US$100 000 or more in the last reported year.
  - Groups funders into precise or broad funding bands (organisations that omit funding bands may be eligible for a C if they name all funders).
- D
  - Names some funders (but only a minority, or not in a systematic way).
- E
  - No or negligible relevant information provided.

### Limitations associated with data disclosures

There were several limitations associated with the data reporting described in the information disclosures on the WHOF website. The Foundation states that ‘These figures do not represent the financial statements of the Foundation, nor have any legal value. Only the audited financial statements approved by the board and published on the website under the financial statements section are the valid and approved financial statements. These figures are posted for information purposes only and will be updated regularly, to ensure transparency of the foundation towards the public’. The WHOF notes that if one donor gave multiple donations to the same purpose, this was combined (see online supplemental appendices), and that donors listed have agreed to be mentioned. If such approval was not given, they are listed as anonymous.

The data was provided as PDF documents (one for each time period) under the ‘governance and reporting’ section of the website. The documents themselves list only the name of the donor, if provided, or state ‘anonymous’ over or under US$100 000. The timing of the donations is not provided beyond which calendar year it falls in. The only information provided regarding earmarking is in a single cell providing the title of the relevant programme or initiative.

Finally, there is some variation in how the data was disclosed year to year. In the first two disclosures (May 2020–December 2021 and January 2022–December 2022), the funding numbers appear to be rounded, but in the final disclosure (January 2023–December 2023), the numbers are not rounded. Finally, in a small number of cases, donations were listed as ‘anonymous’ but not as either being over or under US$100 000. These were included in the overall anonymous donation totals, but not in either subcategory. These limitations should be borne in mind in interpreting any aggregate figures as these may not be accurate and do not represent final audited financial data. The data do, however, allow for an analysis of public disclosures by the WHOF of their funding for information purposes and hence offer an appropriate basis on which to appraise transparency regarding donations.

### Patient and public involvement statement

No patients or members of the public were involved in the design, conduct or reporting of this research.

## Results

### Donation disclosures and donor transparency

From its launch until the end of 2023, the foundation disclosed total donation receipts of US$82 783 930 overall, of which US$39 757 326 (48.0%) was from anonymous donations over US$100 000 (see table 1). In total, US$51 554 203 (62.3%) in anonymous donations were reported. The overall level of income varied by year; however, overall, the proportion of anonymous donations over US$100 000 reported increased year on year.

**Table 1:** Anonymous WHO Foundation donations as a proportion of overall donations disclosed in US dollars

	Anonymous <100K (USD)	Anonymous >100K (USD)	Overall donations received (USD)	% Anonymous >100K	% Anonymous overall
May 2020–December 2021	6 875 000	2 705 000	28 225 400	9.60	33.90
January–December 2022	3 747 983	24 189 233	38 438 331	62.90	72.70
January–December 2023	1 173 894	12 863 093	16 120 199	79.80	87.10
Total	11 796 877	39 757 326	82 783 930	48.00	62.30

USD, US Dollars.

### Donations by donor type

**Figure 1:**
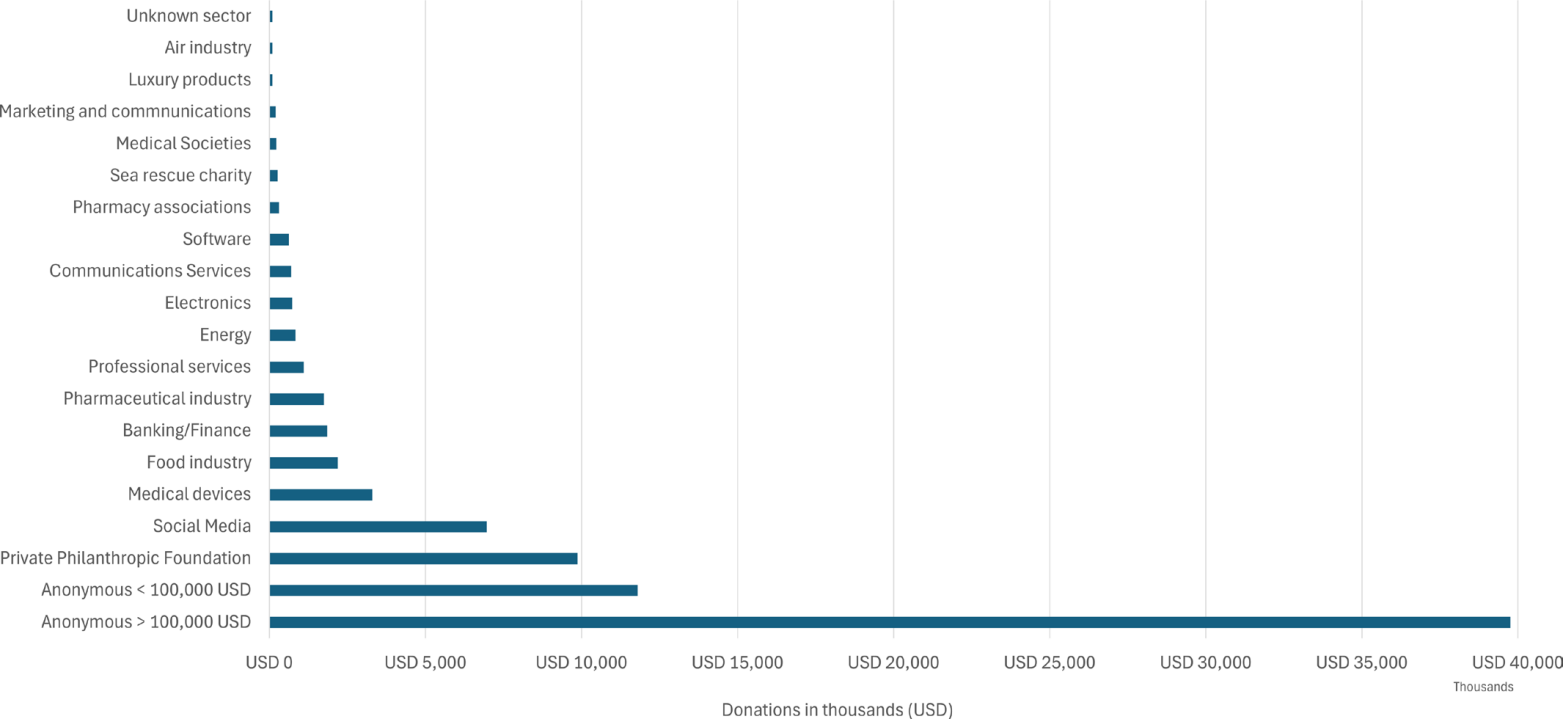
Donations by donor type overall (thousands of dollars) as disclosed between May 2020 and December 2023 by the WHO Foundation. USD, US dollars.

Donations varied by sector, with the largest named donations coming from the private philanthropic sector, including the Gates foundation and other family foundations, followed by social media companies, medical device companies and the banking/finance and pharmaceutical sectors (see figure 1).

### Earmarking to specific programmes

**Figure 2:**
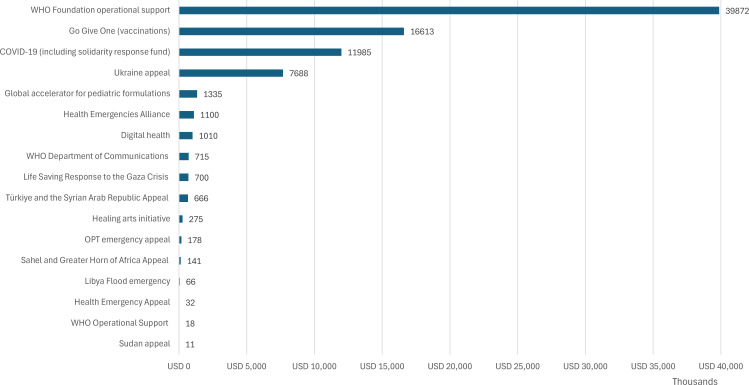
Donations by listed purpose (thousands of dollars) as disclosed between May 2020 and December 2023 by the WHO Foundation. USD, US dollars.

**Figure 3:**
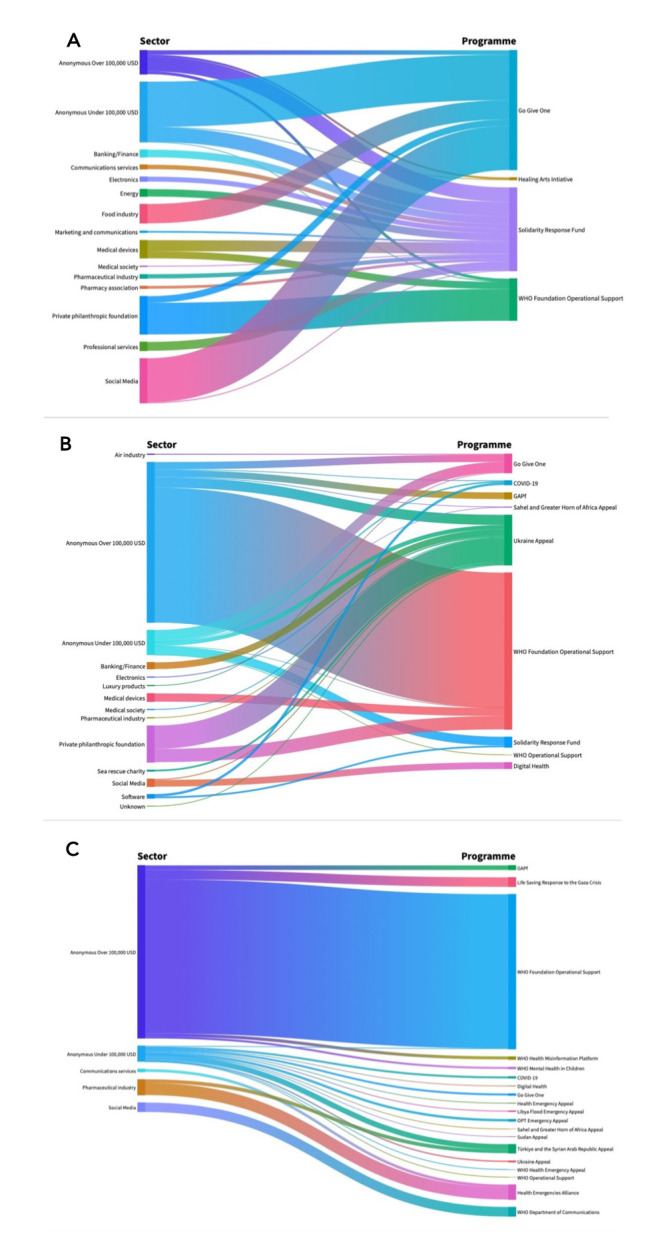
Trends in funding topic over time by sector for (A) May 2020–December 2021, (B) January–December 2022, (C) January–December 2023. GAPf, Global Accelerator for Paediatric Formulations; OPT, Occupied Palestinian Territory; USD, US dollars.

The data disclosed includes a ‘purpose’ linked to each donation. These varied by year consistent with shifts in the specific appeals and programmes launched by the WHOF. Most were linked to a specific appeal (eg, ‘Ukraine appeal’), fund (eg, Solidarity Response Fund) or issue (eg, COVID-19). Overall earmarking for programmes is shown in figure 2 with Sankey diagrams showing earmarking by topic and funders for each time period in figure 3. The largest overall category, by amount donated, was ‘WHO Foundation Operational Support’, which received just under US$40 million over the entire reporting period, representing a majority (approximately 56%) of all funding received by the Foundation to date.

### Transparency rating assessment

Transparency rating assessments were made by reporting period. In each reporting period, the WHOF grouped funders into broad bands (in terms of amounts, funding objectives and sources). In the first year of its operation, according to the approach adopted here, the WHOF would be rated ‘B’ for transparency, as in addition to grouping funders in bands, 16.8% of disclosed donations were anonymous donations over US$100 000. However, in the next two reporting periods, the WHOF would be assessed a ‘D’ for transparency according to the Who Funds You criteria, as it names some funders, but less than 50% by value of donations (anonymous donations over US$100 000 constituted 62.9% of all disclosed donations in January 2022–December 2022, increasing to 79.8% in January 2023–December 2023).

## Discussion

This is the first analysis of funding disclosures by the WHOF by time period, donor type, amount and programme. It identifies several key findings. First, on transparency, we find a reliance on a few, larger, anonymous donations over time, with named donors increasingly in the minority by value. Nearly 80% of funds donated in January–December 2023 were from anonymous sources and in amounts of over US$100 000. In our analysis applying a modified version of Open Democracy’s ‘Who Funds You’ rubric, results suggest that in 2023, the WHOF’s funding transparency is similar to that of the Institute of Economic Affairs or the Legatum Institute in the UK; organisations characterised as ‘dark money’ think tanks.^30^ Second, on earmarking, the data indicate increasing earmarked funding going to the running costs of the WHOF, rather than in support of specific WHO programmes, or flexibly funding the WHO itself. Over time, an increasingly substantial proportion of overall funds received has been earmarked as WHOF operational support, rather than flexible funding, or earmarked funding to support specific WHO initiatives. This included single large anonymous donations to WHOF operational support, including an anonymous donation of over US$11 million in 2023. The analysis also identified a shift in the range of programmes funded during the time periods, reflecting a high (in donations and diversity of donor sources) during the pandemic, with the largest programmes being the COVID-19 vaccine-directed Go Give One and the COVID-19 Solidarity Response Fund, the latter having the most diverse funding pool in terms of range of contributors, towards fewer programmes receiving fewer, smaller donations in the latest disclosures. Critically, while transparency in disclosures decreased, and the diversity of programmes funded narrowed, both the overall donation amounts and those designated to supporting specific WHO initiatives were of a very small and decreasing scale, relative to the size of the WHO biennial budget.

This study has several important limitations. First, our findings are dependent on the accuracy of the disclosure files available on the WHOF website. As noted in the relevant documentation, these are not actual financial statements, but rather, voluntarily disclosed information on donations and linked programmes. Moreover, our analysis was limited by the level of detail provided. While the donations were disclosed according to the calendar year in which they were contributed, they do not include specific time frames or dates. It is therefore not possible to make direct comparisons to financial year reporting of overall contributions or of specific programme activities. Due to a lack of available information, we cannot assess the nature of individual partnership agreements, or the extent to which these were led by or initiated as a result of WHOF priorities, individual donor priorities or a combination thereof. However, in a context of very limited information, this study represents a novel addition to the literature regarding the scale and nature of funding disclosures in what remains a new and contested model of WHO-related funding with potentially significant legitimacy implications for the organisation and for global health. These limitations themselves serve to illustrate the challenges in assessing trends, earmarking and implications for accountability when donation disclosures and the processes by which earmarked funds are negotiated are not fully transparent.

While donor earmarking towards specific WHO priorities decreased over time in favour of WHOF operational costs, our analysis of trends in funded programmes identified large inequalities in such earmarking, with donor emphasis on funding of specific initiatives, such as vaccination, or in specific contexts, such as Ukraine (eg, rather than Libya or Sudan). This raises concerns consistent with wider critiques of vertical approaches, donor conditionality and efficiency of allocation and earmarking in the context of global health initiatives. A long-held critique of the rise of multilateral funds and bilaterally arranged health initiatives has been that donor priorities (often for vertical, specific, technology-focused initiatives with measurable outcomes) take precedent over the national plans, priorities and needs of individual countries,^32^ and emphasise responding reactively to specific high-profile needs over sustained, broad capacity building, a pattern observed most recently in the context of Pandemic Fund deliberations.^33^

The disclosures that are made do in some cases suggest strategic alignment with donor priorities, particularly as it relates to private companies. For example, Meta was disclosed as funding the WHO department of communications and digital health, both areas in which large social media companies have faced public scrutiny due to their potential role in facilitating misinformation, including health misinformation,^34^,^36^ and ongoing debates regarding their role in child and adolescent mental health.^37^ The relevance of concerns around health misinformation and disinformation may have further increased following the announcement from Meta in early 2025 that it was ceasing the use of independent fact-checking in content moderation.^38^ The announcement by the new US administration of withdrawal from the WHO also creates a dynamic in which the WHOF is soliciting and receiving funds from companies such as Meta, while said companies also provided political donations to the political leadership which defunded the WHO itself.^16^

Considering the number and proportion of large anonymous donations, particularly those that go directly to the WHOF, it is difficult to assess the potential for associated conflicts of interest. Such difficulties apply whether in terms of forming longer lasting dynamics of bias or dependence for the WHOF (considering the scale of funding towards WHOF operating costs), or of assessing the potential conflicts of interest from the perspective of donors, particularly considering the broad nature of the programmes of work proposed as future priorities by the WHOF. For example, climate change is among the key priorities announced for WHOF,^19^ but as fossil fuel companies are not mentioned in the foundation’s gift policy, it is not possible to know whether fossil fuel, energy, petrochemical and related industries are specifically excluded from donating, or are donating currently. The BP Foundation was previously a donor to the COVID Solidarity Fund.^2^

Such gaps in disclosure occur in a wider context in which the commercial determinants of health are increasingly acknowledged as key drivers of global health inequalities,^39 40^ not only through the production and sale of harmful products,^41^ but through corporate political activities^13^ and the strategic use of corporate social responsibility initiatives and partnerships as a way of preventing future regulation and therefore protecting future growth.^42^,^45^ In the context of developing actions to address the commercial determinants, DG Tedros has therefore emphasised the need for health governance to ‘focus on imbalances of power’ and prioritise ‘equity, accountability, and precaution’.^46^ Such concerns to ensure effective governance in interactions with the private sector seem hard to reconcile with the WHOF promoting ‘unparalleled access to WHO’ to its corporate partners, and with the strikingly limited levels of transparency regarding donors documented above. Particularly in light of recent changes to global health funding and the recent launch of the ‘One Dollar, One World’ Campaign which explicitly seeks small donations as a way of showing solidarity to the WHO in the wake of the USA withdrawing membership,^47^ there is an urgent need to develop more effective mechanisms for transparency and accountability in such international organisations. The adaptation of existing transparency tools, as in the current study, and their use in grading transparency across greater numbers of such organisations could help identify examples of best practice, as well as areas of greatest concern.

In summary, this analysis of WHOF donor disclosures indicates levels of donor transparency akin to oft-criticised free market think tanks, with attendant risks for both undue influence and/or reputational damage for the WHOF, and by extension the WHO, including in relation to commercial determinants of health. This is particularly the case given the emphasis WHOF places on their close ties with WHO. Such risks appear to have been undertaken for what to date constitute relatively negligible financial benefits to the WHO, much of which follows donor, rather than WHO, priorities.

## Supplementary material

10.1136/bmjgh-2025-018932 online supplemental file 1

10.1136/bmjgh-2025-018932 online supplemental file 2

10.1136/bmjgh-2025-018932 online supplemental file 3

## Data Availability

Data are available upon reasonable request.
