# Supplementary material for: Who funds the WHO Foundation? A transparency analysis of donation disclosures over the first 3 years of its operation
Source: BMJ Glob Health. 2025 Jul 23;10(7):e018932. doi: 10.1136/bmjgh-2025-018932 (PMC12306262; doi:10.1136/bmjgh-2025-018932)
Supplement: online supplemental file 1 [file bmjgh-10-7-s001.pdf]

WHO Foundation		
**Contributions 1 January 2023 to 31 December 2023**		
**Name**	**Purpose**	**Amount USD**
Donations Under 100'000 USD	COVID-19	$149,068
Donations Under 100'000 USD	Digital Health	$10,000
Anonymous	GAPf	$335,000
Donations Under 100'000 USD	Go Give One	$145,009
Foundation S - The Sanofi Collective	Health Emergencies Alliance	$200,000
Merck & Co., Inc.	Health Emergencies Alliance	$200,000
Spotify	Health Emergencies Alliance	$200,000
Pfizer Inc.	Health Emergencies Alliance	$500,000
Donations Under 100'000 USD	Health Emergency Appeal	$31,552
Anonymous	Life Saving Response to the Gaza Crisis	$200,000
Anonymous	Life Saving Response to the Gaza Crisis	$500,000
Donations Under 100'000 USD	Libya Flood Emergency Appeal	$66,474
Donations Under 100'000 USD	OPT Emergency Appeal	$177,597
Donations Under 100'000 USD	Sahel and Greater Horn of Africa Appeal	$14,105
Donations Under 100'000 USD	Sudan Appeal	$11,088
Donations Under 100'000 USD	Türkiye and the Syrian Arab Republic Appeal	$397,885
Foundation S - The Sanofi Collective	Türkiye and the Syrian Arab Republic Appeal	$268,212
Donations Under 100'000 USD	Ukraine Appeal	$104,421
META PLATFORMS, INC.	WHO Department of Communications	$715,000
Donations Under 100'000 USD	WHO Foundation Operational Support	$50,102
Anonymous	WHO Foundation Operational Support	$11,119,760
Anonymous	WHO Foundation Operational Support	$333,333
Donations Under 100'000 USD	WHO Health Emergency Appeal	$3,946
Anonymous Over 100'000 USD	WHO Health Misinformation Platform	$225,000
Anonymous	WHO Mental Health in Children	$150,000
Donations Under 100'000 USD	WHO Operational Support	$12,647
**Overall Total**		**$16,120,198**

For the purpose of this table:
- These figures do not represent the financial statements of the Foundation, nor have any legal value. Only the audited financial statements approved by the board and published on the website under the financial statements section are the valid and approved financial statements. These figures are posted for information purposes only and will be updated regularly, to ensure transparency of the foundation towards the public.
- These figures represent amounts received by the Foundation up to 31 December 2023.
- All amounts are mentioned in USD, but the amounts transferred by contributors may have been received in various currencies. The exchange rate used is the monthly average rate published by the Swiss Federal Tax Administration at the time of receipt of funds by the Foundation.
- Donations are received from individual or legal entities, either through the online tools provided by the Foundation or via bank transfers.
- All donors listed have agreed to be mentioned in this table. If such approval was not given, the donors are mentioned under "anonymous donation". According to the Gift Acceptance Policy of the WHO Foundation, these donors are not anonymous to the Foundation.
- If one donor gave multiple contributions to the same purpose, the funds mentioned from this donor, in this table, are combined.
