# Supplementary material for: Who funds the WHO Foundation? A transparency analysis of donation disclosures over the first 3 years of its operation
Source: BMJ Glob Health. 2025 Jul 23;10(7):e018932. doi: 10.1136/bmjgh-2025-018932 (PMC12306262; doi:10.1136/bmjgh-2025-018932)
Supplement: online supplemental file 2 [file bmjgh-10-7-s002.pdf]

WHO Foundation		
Contributions Through December 31, 2021		
**Name**	**Purpose**	**Amount USD**
Accenture	Solidarity Response Fund	1,000,000
Terumo Corporation	Solidarity Response Fund	1,000,000
EDP – S.A.Energias de Portugal, S.A.	Solidarity Response Fund	840,000
Anonymous Over 100,000 USD*	Solidarity Response Fund	750,000
NOVO BANCO, S.A.	Solidarity Response Fund	560,000
Worten - Equipamentos para o Lar, S.A.	Solidarity Response Fund	560,000
Spotify USA, Inc.	Solidarity Response Fund	500,000
Associação Nacional das Farmácias	Solidarity Response Fund	310,000
Associação Portuguesa de Indústria Farmacêutica	Solidarity Response Fund	280,000
Anonymous Over 100,000 USD*	Solidarity Response Fund	200,000
AON Foundation	Solidarity Response Fund	100,000
West Pharmaceutical Services, Inc.	Solidarity Response Fund	200,000
Publicis, Inc	Solidarity Response Fund	200,000
Royal Bank of Canada Foundation	Solidarity Response Fund	195,000
Anonymous Over 100,000 USD	Solidarity Response Fund	140,000
Anonymous Over 100,000 USD	Solidarity Response Fund	140,000
Align Technology	Solidarity Response Fund	140,000
Thermo Fisher Scientific	Solidarity Response Fund	120,000
Anonymous Over 100,000 USD*	Solidarity Response Fund	115,000
Anonymous Over 100,000 USD*	Solidarity Response Fund	100,000
Anonymous Over 100,000 USD*	Solidarity Response Fund	100,000
Anonymous Over 100,000 USD*	Solidarity Response Fund	100,000
International League of Dermatological Societies	Solidarity Response Fund	100,000
Linkedin	Solidarity Response Fund	100,000
Triumph Int. Limited	Solidarity Response Fund	100,000
Donations Under 100,000 USD	Solidarity Response Fund	1,640,000
Facebook	Go Give One	5,000,000
Nestlé	Go Give One	2,190,400
Anonymous Over 100,000 USD*	Go Give One	500,000
Bill & Melinda Gates Foundation	Go Give One	280,000
ELMA Vaccines and Immunization Foundation	Go Give One	250,000
Greta Thunberg Foundation	Go Give One	120,000
J. Welch	Go Give One	100,000
Donations Under 100,000 USD	Go Give One	5,150,000
Anonymous Over 100,000 USD	Healing Arts Intiative	230,000
Donations Under 100,000 USD	Healing Arts Intiative	45,000
Children's Investment Fund Foundation	WHO Foundation Operational Support	500,000
Mundo Sano Foundation	WHO Foundation Operational Support	3,000,000
Masimo Corporation	WHO Foundation Operational Support	800,000
Anonymous Over 100,000 USD	WHO Foundation Operational Support	330,000
MCJ Amelior Foundation	WHO Foundation Operational Support	100,000
Donations Under 100,000 USD	WHO Foundation Operational Support	40,000
**Overall Total**		**28,225,400**
**Notes:**		

For the purpose of this table:<br>- These figures do not represent the financial statements of the Foundation, nor have any legal value. Only the audited financial statements approved by the board and published on the website under the financial statements section are the valid and approved financial statements. These figures are posted for information purposes only and will be updated regularly, to ensure transparency of the foundation towards the public.<br>- These figures represent amounts received by the foundation up to 31 December 2021. It does not include amounts pledged or paid allocated to the campaigns through fiduciary partners that were not transferred to the Foundation before 31 December 2021.<br>- All amounts are mentioned in USD, but the amounts transferred by contributors may have been received in various currencies. The exchange rate used is the one at the time of the introduction of the said numbers in this table.<br>- All amounts are rounded and do not reflect the precise amount received.<br>- Donations are received from individual or legal entities, either through the online tools provided by the Foundation or via bank transfers.<br>- All donors listed have agreed to be mentioned in this table. If such approval was not given, the donors are mentioned under "anonymous donation". According to the Gift Acceptance Policy of the WHO Foundation, the donors are not anonymous to the Foundation.<br>- When one donor gave multiple contributions to the same purpose, the funds mentioned from this donor, in this table, are combined.<br>*These donations are mentioned "Anonymous", however, these contributions were donated through our fiduciary partners and we are in the process of seeking approval of donors to be mentioned in this table.
