# Supplementary material for: Who funds the WHO Foundation? A transparency analysis of donation disclosures over the first 3 years of its operation
Source: BMJ Glob Health. 2025 Jul 23;10(7):e018932. doi: 10.1136/bmjgh-2025-018932 (PMC12306262; doi:10.1136/bmjgh-2025-018932)
Supplement: online supplemental file 3 [file bmjgh-10-7-s003.pdf]

WHO Foundation		
**Contributions 1 January 2022 to 31 December 2022**		
**Name to be Published**	**Purpose**	**Amount USD**
Pegasystems Inc.	COVID-19	$364,423
Donations Under 100'000 USD	COVID-19	$201,588
Anonymous Over 100'000 USD	COVID-19	$101,450
META PLATFORMS, INC.	Digital Health	$1,000,000
Anonymous	GAPf	$1,000,000
Anonymous	Go Give One	$1,000,000
Donations Under 100'000 USD	Go Give One	$1,578,089
Anonymous Over 100'000 USD	Go Give One	$100,000
DFS Group	Go Give One	$100,000
Southwest Airlines	Go Give One	$100,000
Donations Under 100'000 USD	WHO Operational Support	$4,980
Anonymous Over 100'000 USD	Sahel and Greater Horn of Africa Appeal	$122,450
Donations Under 100'000 USD	Sahel and Greater Horn of Africa Appeal	$4,712
Donations Under 100'000 USD	Solidarity Response Fund	$1,312,927
IBM Corporation	Solidarity Response Fund	$265,304
The Patchwork Collective	Ukraine Appeal	$1,022,000
Anonymous Over 100'000 USD	Ukraine Appeal	$1,022,000
Schwartz Family Charitable Fund	Ukraine Appeal	$1,000,000
Sea Grape Foundation	Ukraine Appeal	$1,000,000
UBS OPTIMUS FOUNDATION	Ukraine Appeal	$1,000,000
Anonymous Over 100'000 USD	Ukraine Appeal	$500,000
David Karp and Samantha McManus	Ukraine Appeal	$500,000
Donations Under 100'000 USD	Ukraine Appeal	$625,922
Civilfleet-Support e.V.	Ukraine Appeal	$266,873
Micron Technology	Ukraine Appeal	$175,000
META PLATFORMS, INC	Ukraine Appeal	$150,000
Royal College of Nursing	Ukraine Appeal	$121,588
Progress	Ukraine Appeal	$100,000
West Pharmaceutical Services	Ukraine Appeal	$100,000
Anonymous Over 100'000 USD	WHO Foundation Operational Support	$20,000,000
Masimo Corporation	WHO Foundation Operational Support	$1,236,000
The Bill & Melinda Gates Foundation	WHO Foundation Operational Support	$1,000,000
Mundo Sano Foundation	WHO Foundation Operational Support	$999,927
Anonymous Over 100'000 USD	WHO Foundation Operational Support	$343,333
Donations Under 100'000 USD	WHO Foundation Operational Support	$19,765
**Overall Total**		**$38,438,331**

Notes:

For the purpose of this table:
- These figures do not represent the financial statements of the Foundation, nor have any legal value. Only the audited financial statements approved by the board and published on the website under the financial statements section are the valid and approved financial statements. These figures are posted for information purposes only and will be updated regularly, to ensure transparency of the foundation towards the public.
- These figures represent amounts received by the foundation up to 31 December 2022.
- All amounts are mentioned in USD, but the amounts transferred by contributors may have been received in various currencies. The exchange rate used is the one at the time of the introduction of the said numbers in this table.
- Donations are received from individual or legal entities, either through the online tools provided by the Foundation or via bank transfers.
- All donors listed have agreed to be mentioned in this table. If such approval was not given, the donors are mentioned under "anonymous donation". According to the Gift Acceptance Policy of the WHO Foundation, these donors are not anonymous to the Foundation.
- If one donor gave multiple contributions to the same purpose, the funds mentioned from this donor, in this table, are combined.
